# Dataset of volatile compounds from flowers and secondary metabolites from the skin pulp, green beans, and peaberry green beans of robusta coffee

**DOI:** 10.1016/j.dib.2020.105219

**Published:** 2020-01-31

**Authors:** Hafsah Hafsah, Iriawati Iriawati, Tati Suryati Syamsudin

**Affiliations:** School of Life Sciences and Technology, Institut Teknologi Bandung, West Java, Indonesia

**Keywords:** Robusta coffee, Solid-phase microextraction, Gas chromatography-mass spectrometry, Metabolites, Wet-hulled

## Abstract

We obtained data regarding the metabolites from flowers, the skin pulp, green beans and peaberry green beans of the robusta coffee plant (*Coffea canephora*). The beans were processed using a wet-hulled method. The volatile compounds from the flowers were extracted using a solid-phase microextraction. Secondary metabolites from the skin pulp, green beans, and peaberry green beans were extracted by a maceration method using methanol as a solvent. The separation and identification of metabolites were conducted using gas chromatography-mass spectrometry. The flower's volatile compounds were identified by matching the generated spectra with the NIST14 library as a reference, whereas the metabolites in the skin pulp, green beans, and peaberry green beans were identified using the WILLEY09TH library as a reference. The identified volatile compounds in flowers have been listed in Table 1, and the identified skin pulp, green bean, and peaberry green bean metabolite compounds have been listed in Table 2.

**Table:** Specifications Table

Subject	Agriculture and Biological Science
Specific subject area	Biochemical diversity. The data provide insights into the metabolic profiles of flowers and green beans from the robusta coffee plant, demonstrating a biochemical diversity
Type of data	Table
How data were acquired	The volatile compounds from the flowers of the robusta coffee plant were extracted by solid-phase microextraction (SPME) and analyzed using gas chromatography-mass spectrometry (GC-MS; GC: 7890A, MS: 5975C, Agilent Technologies, Inc., CA, USA). The skin pulp, green beans, and peaberry green beans were extracted using a maceration method with a methanol-based solvent and were analyzed using GC-MS (GC: 6890N, MS: 5973, Agilent Technologies, Inc.)
Data format	Raw
Parameters for data collection	The five parts of the robusta coffee plant, i.e., flowers, skin pulp of beans, skin pulp of peaberries, green beans, and peaberry green beans, were analyzed. All parts were collected from *Coffea canephora* cv. Tugusari.
Description of data collection	All samples was collected from a low land robusta coffee orchard (at 680 m above sea level). Only fresh anthesis flowers and mature coffee fruits (cherries) were picked for analysis. The beans were processed using a wet-hulled method. Volatile compounds in the coffee flowers were analyzed, and the profiles of secondary metabolites were analyzed from the skin pulp and beans. Floral volatile compounds were extracted by SPME, whereas metabolites from the skin pulp and beans were extracted by maceration with methanol as a solvent. Identification of volatile compounds, determination of retention times, and measurement of peak areas were performed using GC-MS.
Data source location	Jember District, East Java – Indonesia at South Latitude 08° 13′ and East Longitude 113° 55’.
Data accessibility	With the article

**Table:** 

**Value of the Data** • These data contributed to our understanding of volatile compounds in the flowers and secondary metabolites in the skin pulp and beans from the robusta coffee plant, respectively. • The data are important for coffee entrepreneurs or coffee merchants (torrefacteurs), researchers, academics, farmers, and policymakers involved in coffee plantation management. • Elucidation of the volatile compounds of the robusta coffee flowers is important for improving our understanding of the chemical/metabolic diversity and recognition of potential insects as pollinators that may contribute to the pollination of the robusta flowers. • Information on the secondary metabolites of the skin pulp may facilitate the development of insect attractants or repellents, which may contribute to pest control programs. • The data on the metabolites of robusta beans may be used to understand the effects of different postharvest treatments (such as wet-hulling) on the quality and flavors of the robusta coffee. • The data on metabolites from the skin pulp may also be important for other uses of the beans, such as in teas or infusions.

## 1 Data description

These raw data include information on the volatile compounds of the robusta coffee flowers and the profiles on the secondary metabolites of the skin pulp, green beans, and peaberry green beans of the robusta coffee. The raw data have been provided in a Microsoft Excel Worksheet (Table 1, Table 2) and have been presented with retention times, identified volatile compounds, and peak areas.

**Table 1:** Retention times, identified compounds, and relative peak areas (%) from the GC chromatogram of Robusta coffee flower.

Retention Time (min.)(n = 3)				Compounds	Relative Peak Area (%) (n = 3)		
1		2	3		1	2	3
1.794			1.794	Ethanol	3.62	n.d	3.3
		4.309		Cyclobutylcarboxylic acid	n.d	0.34	n.d
6.450			6.438	Butanoic acid, 3-methyl-, ethyl ester	0.17	n.d	0.27
		6.515		1H-Indole, 5-methyl-2-phenyl-	n.d	0.05	n.d
		7.009		1-Butanol	n.d	0.04	n.d
			7.693	Carbamic acid, methyl-, ethyl ester	n.d	n.d	0.02
			8.073	2-Heptanol	n.d	n.d	0.16
8.076				2-Pentadecanol	0.11	n.d	n.d
		8.079		4-Methyl-2-hexanol	n.d	0.04	n.d
10.303		10.232	10.291	Benzaldehyde	0.23	0.13	0.10
11.308		11.314	11.290	β-Myrcene	0.67	0.73	0.54
12.574		12.574	12.568	d-Limonene	0.15	0.18	0.12
13.228		13.335	13.228	Benzyl alcohol	4.92	3.23	3.09
14.073		14.655		Ethyl 2-(5-methyl-5-vinyltetrahydrofuran-2-yl)propan-2-yl carbonate	0.69	0.53	n.d
		14.085	14.090	trans-Linalool oxide (furanoid)	n.d	0.60	0.48
14.810		14.822	14.804	Benzoic acid, methyl ester	2.70	1.15	1.62
15.440		15.541	15.458	Linalool	22.29	27.03	22.23
15.642		15.726	15.654	Phenylethyl Alcohol	0.20	0.17	0.20
15.993		16.035	15.999	2,4,6-Octatriene, 2,6-dimethyl-, (E,Z)-	0.13	0.15	0.10
16.754		16.962		Benzyl nitrile	14.56	0.04	n.d
			16.944	3,6-Dimethyl-2,3,3a,4,5,7a-hexahyd robenzofuran	n.d	n.d	0.05
			17.135	Benzene, (isocyanomethyl)-	n.d	n.d	1.37
17.135		17.141		Isoneral	0.04	0.04	n.d
17.343		17.349	17.343	Benzoic acid, ethyl ester	1.50	0.88	1.37
17.498				Deltacyclene	2.03	n.d	n.d
		17.509	17.491	5H-1-Pyrindine	n.d	1.74	1.54
17.670		17.676	17.658	Indole	1.50	1.33	1.17
17.896		17.908	17.896	α-Terpineol	0.02	0.02	0.01
		18.009		Methyl salicylate	n.d	0.29	n.d
18.027			18.021	Dodecane	0.19	n.d	0.18
18.687		18.693	18.687	6-Octen-1-ol, 7-methyl-3-methylene	0.03	0.03	0.02
19.020		19.055	19.020	2,6-Octadien-1-ol, 3,7-dimethyl-, (Z)-	1.96	2.62	1.84
19.127		19.406	19.127	3,6-Octadien-1-ol, 3,7-dimethyl-,(Z)-	0.20	0.10	0.19
19.317		19.341	19.317	2,6-Octadienal, 3,7-dimethyl-, (Z) Citral	0.15	0.22	0.12
19.418			19.424	Benzeneacetic acid, ethyl ester	0.24	n.d	0.38
19.781		19.816	19.781	Geraniol	1.76	2.07	1.59
20.155			20.155	2,6-Octadienal, 3,7-dimethyl-, (E)	0.29	n.d	0.31
		20.173		2,6-Octadienal, 3,7-dimethyl-	n.d	0.38	n.d
20.322				2,6-Octadien-1-ol, 3,7-dimethyl-, (Z)-	0.02	n.d	n.d
			20.405	2,6-Octadienoic acid, 3,7-dimethyl -, methyl ester	n.d	n.d	0.01
20.548				5-Tridecene, (E)-	0.04	n.d	n.d
		20.554	20.548	6-Tridecene, (E)-	n.d	0.04	0.03
21.018		21.018	21.023	Tridecane	5.50	5.01	5.46
21.291		21.434	21.291	Indole	0.02	0.01	0.02
21.582			21.582	trans-Geranic acid methyl ester	0.10	n.d	0.11
22.153		22.153	22.147	Methyl anthranilate	0.76	0.65	0.58
22.278			22.278	Benzenepropanoic acid, ethyl ester	0.14	n.d	0.11
		23.087		5-Tetradecene, (E)-	n.d	0.02	n.d
			23.087	3-Tetradecene, (Z)-	n.d	n.d	0.01
23.081		23.206		7-Tetradecene, (Z)-	0.01	0.00	n.d
23.331		23.331	23.330	3-Tetradecene, (E)-	0.03	0.03	0.03
23.551		23.551	23.550	Tetradecane	0.41	0.38	0.43
23.872		23.872	23.872	Benzoic acid, 2-(methylamino)-, methyl ester	0.01	0.01	0.01
		24.175		Caryophyllene	n.d	0.00	n.d
25.754		25.751	25.750	1-Tridecene	4.56	4.36	4.60
25.858				n-Tridecan-1-ol	1.07	n.d	n.d
		25.858	25.857	Cyclopentadecane	n.d	1.00	1.09
26.583		26.583	26.607	Pentadecane	18.68	17.12	19.24
28.064				Succinic acid, di(3-methylbut-3-enyl) ester	0.00	n.d	n.d
			28.069	Succinic acid, hex-4-yn-3-yl 3-methylbut-3-en-1-yl ester	n.d	n.d	0.00
		28.064		Supraene	n.d	0.00	n.d
28.301				Benzene, [(2,2-dimethylcyclopropyl)methyl]-	0.00	n.d	n.d
28.593			28.593	(3E,7E)-4,8,12-Trimethyltrideca-1,3,7,11-tetraene	0.07	n.d	0.09
		28.593		Squalene	n.d	0.00	n.d
29.134		29.128	29.134	Hexadecane	0.07	0.05	0.06
29.532				Pentadecanal-	0.03	n.d	n.d
		29.532	29.532	Tetradecanal	n.d	0.05	0.02
31.072		31.072	31.072	6,9-Heptadecadiene	0.27	0.05	0.28
31.209			31.209	1,4-Cyclooctadiene, (Z,Z)-	0.17	n.d	0.16
		31.209		Tricyclo[4.2.1.1(2,5)]decan-3-ol	n.d	0.18	n.d
31.411		31.399	31.411	8-Heptadecene	3.51	2.86	3.34
31.893		31.881	31.893	Heptadecene	1.21	0.95	1.22
		32.089		Pentadecafluorooctanoic acid, dodecyl ester	n.d	0.01	n.d
32.095			32.243	Cyclopentane, pentyl-	0.01	n.d	0.00
32.244				3,6-Octadienal, 3,7-dimethyl-	0.01	n.d	n.d
		32.244		Cyclohexene, 4-methyl-	n.d	0.01	n.d
		32.434		3,4-Octadiene, 7-methyl-	n.d	0.01	n.d
32.440				Cyclododecene, (E)-	0.01	n.d	n.d
			32.440	E,E-10,12-Hexadecadienal	n.d	n.d	0.00
33.260		33.260	33.266	Z,Z-10,12-Hexadecadienal	0.00	0.00	0.00
		34.241	34.247	Octadecane	n.d	0.03	0.03
34.247				Dodecane, 2,6,11-trimethyl-	0.03	n.d	n.d
34.396		34.396		cis-11-Hexadecenal	0.00	0.01	n.d
			34.396	13-Octadecenal, (Z)-	n.d	n.d	0.00
34.669		34.670		Tetradecanal	0.16	0.18	n.d
			34.669	Hexadecanal	n.d	n.d	0.17
34.872				Isopropyl myristate	0.00	n.d	n.d
			35.692	9-Tetradecen-1-ol, acetate, (Z)-	n.d	n.d	0.01
35.698				1,9-Tetradecadiene	0.01	n.d	n.d
35.787				Z-1,6-Tridecadiene	0.01	n.d	n.d
		35.787	35.787	Cyclododecene	n.d	0.01	0.01
35.924				9,12,15-Octadecatrienal	0.01	n.d	n.d
		35.924	35.924	9,12-Octadecadien-1-ol, (Z,Z)	n.d	0.01	0.01
36.019		36.138		9-Nonadecene	0.02	0.00	n.d
		36.019	36.019	Z-5-Nonadecene	n.d	0.02	0.02
			36.120	Cyclotetradecane	n.d	n.d	0.02
36.126				1-Nonadecene	0.02	n.d	n.d
36.501		36.501	36.507	Nonadecane	0.47	0.46	0.55
38.546		38.540	38.540	Eicosane	0.01	0.01	0.01
40.479		40.479	40.479	Heneicosane	0.02	0.02	0.06
43.594		43.600	43.594	9-Tricosene, (Z)-	0.00	0.00	0.00

**Table 2:** Retention times, identified compounds, and relative peak areas (%) in the GC chromatogram from skin-pulp, pea berry skin-pulp, green bean and pea berry green bean.

Retention Time (min.)(n = 2)								Compounds	Relative Peak Area (%) (n = 2)							
Skin-Pulp		Pea berry skin-pulp		Green bean		Pea berry green bean			Skin-Pulp		Pea berry skin-pulp		Green bean		Pea berry green bean	
1	2	1	2	1	2	1	2		1	2	1	2	1	2	1	2
1.919	1.989	1.728	2.032		2.005	1.935	2.162	Acetic acid	n.d	4.44	n.d	9.45	n.d	0.36	1.18	0.77
		2.187						1,2,3,4-Butanetetrol, [S-(R*,R*)]-	n.d	n.d	0.57	n.d	n.d	n.d	n.d	n.d
2.330								3,4-Furandiol, tetrahydro-, trans-	0.46	n.d	n.d	n.d	n.d	n.d	0.39	n.d
						2.345		3-Ethoxy-1,2-propanediol	n.d	n.d	n.d	n.d	n.d	n.d	0.39	n.d
	2.379							Glycerin	n.d	0.24	n.d	n.d	n.d	n.d	n.d	n.d
		2.387						Pentanal	n.d	n.d	0.3	n.d	n.d	n.d	n.d	n.d
				2.542	2.530	2.473	2.630	Pyridine	n.d	n.d	n.d	n.d	0.66	0.28	1.35	0.57
	2.531							1,2-Benzenediol, 4-(2-amino-1-hydroxyethyl)-, (R)-	n.d	0.24	n.d	n.d	n.d	n.d	n.d	n.d
			2.787					Cyclobutanol	n.d	n.d	n.d	0.42	n.d	n.d	n.d	n.d
2.742		2.638						Butyric acid hydrazide	1.40	n.d	1.08	n.d	n.d	n.d	n.d	n.d
3.119								Pyridine, 1-oxide	0.51	n.d	n.d	n.d	n.d	n.d	n.d	n.d
			3.120					Furfural	n.d	n.d	n.d	0.46	n.d	n.d	n.d	n.d
3.471		3.401	3.441					2-Furanmethanol	0.77	n.d	0.88	0.90	n.d	n.d	n.d	n.d
4.771			7.282					2,3-Dihydro-3,5-dihydroxy-6-methyl-4H-pyran-4-one	2.10	n.d	n.d	4.16	n.d	n.d	n.d	n.d
5.166	5.080	5.135	5.123	5.098		5.098	5.071	Phenol	1.59	1.46	1.15	1.47	0.39	n.d	1.31	0.52
						5.414		2-Oxabicyclo[3.2.0]hepta-3,6-diene	n.d	n.d	n.d	n.d	n.d	n.d	0.6	n.d
6.215	6.194	6.141	6.216					Furaneol	0.80	0.29	0.81	1.05	n.d	n.d	n.d	n.d
6.410						6.372	6.315	Phenol, 2-methoxy-	1.41	n.d	n.d	n.d	n.d	n.d	0.77	0.44
						6.577		Pentanal	n.d	n.d	n.d	n.d	n.d	n.d	0.48	n.d
		7.247	8.019					4H-Pyran-4-one, 2,3-dihydro-3,5-dihydroxy-6-methyl-	n.d	n.d	4.85	0.63	n.d	n.d	n.d	n.d
	7.252							2,3-Dihydro-3,5-dihydroxy-6-methyl -4H-pyran-4-one	n.d	3.2	n.d	n.d	n.d	n.d	n.d	n.d
7.962								Benzoic acid	0.60	n.d	n.d	n.d	n.d	n.d	n.d	n.d
		8.244						Ether, hexyl isopropyl	n.d	n.d	0.78	n.d	n.d	n.d	n.d	n.d
8.378	8.323	8.413	8.336	8.440	8.604	8.449	8.453	1,2-Benzenediol	9.18	7.71	5.36	6.62	1.07	0.6	2.59	1.23
	8.587							6-methoxy-2,2-dimethylbenzo[h]chromene	n.d	2.57	n.d	2.42	n.d	n.d	n.d	n.d
				9.004		8.988	8.917	Phenol, 4-ethyl-2-methoxy-	n.d	n.d	n.d	n.d	0.15	n.d	0.52	0.38
	9.411		9.424	9.466	9.427	9.483	9.411	2-Methoxy-4-vinylphenol	n.d	0.261	n.d	0.21	5.34	1.77	3.96	1.5
	9.966	9.779	9.983		9.731	9.620	9.779	Hydroquinone	n.d	1.3	6.52	1.25	n.d	1.41	5.03	2.86
9.726			9.684					1,2-Benzenediol, 4-methyl-	7.87	n.d	n.d	6.93	n.d	n.d	n.d	n.d
						10.749		1,3-Dimethyl-5-(isopropyl)pyrazole	n.d	n.d	n.d	n.d	n.d	n.d	0.24	n.d
10.810							10.746	4-Ethylcatechol	0.43	n.d	n.d	n.d	n.d	n.d	n.d	0.14
						11.578		2,1,3-Benzothiadiazole	n.d	n.d	n.d	n.d	n.d	n.d	1.05	n.d
							11.613	1-phenyl-2-(3,5,6-trimethylpyrazin-2-yl)ethanol	n.d	n.d	n.d	n.d	n.d	n.d	n.d	0.32
							14.618	Tetradecanoic acid	n.d	n.d	n.d	n.d	n.d	n.d	n.d	0.76
15.540	15.285	14.292	15.047	14.501	15.297			Quinic acid	21.80	9.16	28.23	27.43	1.25	13.04	n.d	n.d
					15.575			Octyl thioglycolate	n.d	n.d	n.d	n.d	n.d	0.71	n.d	n.d
15.891	15.805	15.883	15.801	16.160	16.117	16.280	16.607	Caffeine	16.02	4.51	10.26	5.52	79	51.15	52.46	74.88
			16.096	16.211				Hexadecanoic acid, methyl ester	n.d	n.d	n.d	1.08	1.59	n.d	n.d	n.d
16.715	16.664	16.676	16.647		16.689	16.775	16.785	n-Hexadecanoic acid	15.14	6.04	3.41	8.05	n.d	5.4	2.20	2.63
			17.730					12-Octadecenoic acid, methyl ester	n.d	n.d	n.d	0.32	n.d	n.d	n.d	n.d
	18.250			17.852	17.695	17.818	17.713	9,12-Octadecadienoic acid (Z,Z)-, methyl ester	n.d	4.61	n.d	n.d	1.13	0.77	0.18	0.38
					17.937			Methyl stearate	n.d	n.d	n.d	n.d	n.d	0.16	n.d	n.d
				18.126				Methyl 16-methyl-heptadecanoate	n.d	n.d	n.d	n.d	0.15	n.d	n.d	n.d
					18.263	18.468	18.285	9,12-Octadecadienoic acid (Z,Z)-,	n.d	n.d	n.d	n.d	n.d	3.71	1.43	1.58
		18.297						1,2-Epoxy-1-vinylcyclododecene	n.d	n.d	0.57	n.d	n.d	n.d	n.d	n.d
	18.302		18.290					9,12,15-Octadecatrienoic acid, (Z,Z,Z)-	n.d	2.02	n.d	1.58	n.d	n.d	n.d	n.d
	18.420		18.407		18.423			Octadecanoic acid	n.d	2.88	n.d	2.39	n.d	1.73	n.d	0.09
	20.041				20.044		20.054	Eicosanoic acid	n.d	0.45	n.d	n.d	n.d	0.38	n.d	0.14
	21.211							Palmitoyl chloride	n.d	0.65	n.d	n.d	n.d	n.d	n.d	n.d
21.319		21.337	21.216					Glycerol 1-palmitate	0.46	n.d	0.47	0.44	n.d	n.d	n.d	n.d
		21.666						Nonadecanoic acid	n.d	n.d	0.59	n.d	n.d	n.d	n.d	n.d
21.787	21.658		21.663			23.195		7,9-Dimethoxy-8-isopropyl-4-methyl -1H-phenalen-1-one	0.53	1.85	n.d	0.73	n.d	n.d	4.92	n.d
				22.571	21.219	22.563	21.216	Hexadecanoic acid, 2-hydroxy-1-(hydroxymethyl)ethyl ester	n.d	n.d	n.d	0.45	0.93	1.75	0.61	0.33
						22.657		Benzene, (1-methyl-1-butenyl)-	n.d	n.d	n.d	n.d	n.d	n.d	0.28	n.d
22.672								(R)-(-)-14-Methyl-8-hexadecyn-1-ol	0.61	n.d	n.d	n.d	n.d	n.d	n.d	n.d
			22.560					2-Methyl-Z,Z-3,13-octadecadienol	n.d	n.d	n.d	0.59	n.d	n.d	n.d	n.d
		22.685						1,3,12-Nonadecatriene	n.d	n.d	0.64	n.d	n.d	n.d	n.d	n.d
					23.370			5,9,13-Pentadecatrien-2-one, 6,10,14-trimethyl-, (E,E)-	n.d	n.d	n.d	n.d	n.d	0.27	n.d	n.d
						24.785		9,12-Octadecadienoyl chloride, (Z, Z)	n.d	n.d	n.d	n.d	n.d	n.d	1.03	n.d
				24.794	22.576			9,12-Octadecadienoic acid (Z,Z)-, 2-hydroxy-1-(hydroxymethyl)ethyl ester	n.d	n.d	n.d	n.d	0.99	4.24	n.d	n.d
					24.987			6-methyl-2,3-dihydro-1H-imidazo[1,2-a]pyrimidin-7-one	n.d	n.d	n.d	n.d	n.d	0.15	n.d	n.d
			25.803					1H-Purin-2-amine, 6-methoxy-	n.d	n.d	n.d	0.94	n.d	n.d	n.d	n.d
25.975					25.789			Vitamin E	0.78	n.d	n.d	n.d	n.d	0.51	n.d	n.d
27.111	26.899	27.115	26.891		26.877			Ergost-5-en-3-ol, (3.β.)-	0.45	1.07	0.87	0.62	n.d	0.32	n.d	n.d
			27.233					2-[(4-tert-butylphenyl)methyl]propane-1,3-diol	n.d	n.d	n.d	0.35	n.d	n.d	n.d	n.d
	27.242						27.242	(24S)-ergosta-5,22(E)-dien-3β-ol	n.d	0.58	n.d	n.d	n.d	n.d	n.d	0.23
							27.780	4,7-Methano-1H-indene, octahydro-	n.d	n.d	n.d	n.d	n.d	n.d	n.d	0.13
28.186	27.953	28.186			27.917	32.445		β-Sitosterol	1.61	2.82	2.50	n.d	2.02	0.52	0.73	n.d
				29.025		29.000		β-Tocopherol	n.d	n.d	n.d	n.d	0.20	n.d	0.22	n.d
				30.154		30.111		dl-α-Tocopherol	n.d	n.d	n.d	n.d	0.33	n.d	0.34	n.d
						31.274		Campesterol	n.d	n.d	n.d	n.d	n.d	n.d	0.23	n.d
				31.718		31.676		Stigmasterol	n.d	n.d	n.d	n.d	0.73	n.d	0.54	n.d
				32.496				γ-Sitosterol	n.d	n.d	n.d	n.d	0.69	n.d	n.d	n.d
						34.488		9,19-Cyclolanostan-3-ol, 24-methylene-, (3β)-	n.d	n.d	n.d	n.d	n.d	n.d	0.13	n.d
				32.770				Fucosterol	n.d	n.d	n.d	n.d	0.33	n.d	n.d	n.d

## 2 Experimental design, materials, and methods

a. Preparation and analysis of the flower samples

All samples were collected from a low land robusta coffee orchard (at 680 m above sea level). Only fresh anthesis flowers were picked for analysis. Three sets of samples (10 fresh anthesis of the robusta coffee flowers, approximately 1.5 g) were placed in 22-mL clear glass bottles for SPME with PTFE/Silicon septa (Supelco Co., Bellefonte, PA, USA). After 24 h, the flowers were extracted and identified by an SPME connected with a GC-MS (GC: 7890A, MS: 5975C, Agilent) following the procedure reported by Syamsudin et al. [1]. Coffee flowers in the SPME bottles were extracted at 40 °C for 45 min. The extract was injected into a gas chromatograph at 250 °C for 5 min using a spitless mode. The oven temperature was initially set to 50 °C and held for 5 min. Then, the temperature was increased to 150 °C (5 °C/min for 2 min) and then to 250 °C (5 °C/min for 5 min). An HP-5MS (30 m × 250 μm × 0.25 μm) column was used to separate the volatile compounds with helium as the carrier gas injected at 0.8 mL/min. The flower volatile compounds were identified by matching the generated spectra with the spectra in the NIST14 library as references.

b. Preparation and analysis of skin pulp and bean samples

Only red fruits of the coffee plants were included in this analysis. All fruits (‘normal’ beans and peaberries) were picked by hand from the orchard and processed using the wet-hulled method. Washed coffee fruits were then peeled to obtain the skin pulp. The seeds (beans) were fermented anaerobically for 12 h in a sealed plastic bag, and the mucilage was then washed away. The skin pulp and beans were dried in a screen house (temperature: 32.90 °C ± 5.87 °C; relative humidity: 46.14% ± 16.26%) for 3 weeks. According to standard agricultural practices, the skin pulp and beans were kept at room temperature (24–26 °C). Then, the skin pulp and beans were freeze dried for 24 h and crushed using a coffee grinder (Cyprus International 200W). Next, 100 mg powder of each sample (skin pulp beans, skin pulp peaberry beans, green beans, and peaberry green beans) was macerated for 4 × 24 h using methanol. All extracted samples were evaluated in duplicate. The extract was filtered (Whatman paper No. 91) then evaporated with a rotary evaporator. The macerated samples were redissolved in 1 mL methanol (chromatography-grade; Merck LiChrosolv Reag. Ph Eur). Before injecting into the GC-MS, the sample was filtered with a 0.22-μm/25 mm PTFE filter syringe (Axiva Sichem Biotech Pvt. Ltd. India).

Extraction of the skin pulp and beans was performed with the Sunarharum method [2] using GC-MS with some modifications. The extract was injected into the GC-MS (GC: 6890N, MS: 5973; Agilent Technologies Inc.) at 290 °C using a split mode. The initial oven temperature for green bean and peaberry green bean extracts was set to 50 °C. The temperature was increased to 220 °C (10 °C/min) and then to 290 °C (5 °C/min for 10 min). For ‘normal’ bean skin pulp and peaberry skin pulp extracts, the temperature was increased to 290 °C at 10 °C/min for 15 min. An HP-5MS (30 m × 250 μm × 0.25 μm) column was used to separate volatile compounds with helium as the carrier gas at 1.0 mL/min. The compounds were identified by matching the generated spectra with the spectra from the WILLEY09TH library database.
